# *RPGRorf15* nanopore long-read sequencing improves retinitis pigmentosa molecular diagnosis for men and women

**DOI:** 10.1007/s00439-025-02807-0

**Published:** 2026-02-13

**Authors:** Manon Fabard, Aurore Devos, Anaïs F. Poncet, Jean-Pascal Meneboo, Martin Figeac, Céline Villenet, Isabelle Drumare, Sabine Defoort-Dhellemmes, Isabelle Meunier, Xavier Zanlonghi, Olivier Grunewald, Vincent Huin, Claire Lecigne, Vasily Smirnov, Claire-Marie Dhaenens

**Affiliations:** 1 University of Lille, Inserm, CHU Lille, U1172-LilNCog-Lille Neuroscience & Cognition, 59000 Lille, France; 2 University of Lille, CNRS, Inserm, CHU Lille, Institut Pasteur de Lille, US 41-UAR 2014-PLBS, 59000 Lille, France; 3 Service d’Exploration de la Vision et de Neuro-Ophtalmologie, CHU Lille, Hôpital Salengro, 59037 Lille, France; 4 Institute for Neurosciences of Montpellier (INM), University of Montpellier, INSERM, Montpellier, France; 5 National Reference Center for Inherited Sensory Diseases, University of Montpellier, CHU, Montpellier, France; 6 Service d’Ophtalmologie, Centre de compétence maladie rare, Centre Hospitalier Universitaire de Rennes, Rennes, Bretagne France

## Abstract

*RPGR (Retinitis Pigmentosa GTPase Regulator*) is the main gene involved in X-linked retinitis pigmentosa (RP) and up to 28% of sporadic RP. Pathogenic variants are mostly located in its retina-specific transcript, *RPGRorf15*, especially in the terminal exon named *open reading frame 15 (ORF15)*, which is a highly repeated region. Because of this complex sequence, first- and second-generation short-read sequencing are difficult, both for male patients and for affected and relative females, resulting in numerous unresolved cases. Long-read sequencing appears to be the most appropriate to counter this problem. Using Oxford Nanopore Technology sequencing, we analysed 194 patients with retinal dystrophy (RD) (142 men and 52 women) suspected to be either X-linked (*n* = 42) or autosomal (*n* = 152), and for whom first-line sequencing of 230 retinal dystrophy genes, including *RP2* and *RPGRex1-19*, was negative. Pathogenic *ORF15* variants were found in 27 patients (13.9%), 21 men and 6 women. Of the 42 XLRD patients, 15 were carriers (35.7%), nine men and six women, most of them had rod-cone dystrophy. Of the 152 patients with isolated RD, only 12 carried *ORF15* variants (7.9%),all male patients, most of them had cone-rod dystrophy (41.7%). We demonstrate the efficacy of this technology to diagnose male cases with a family history of X-linked RD, as well as sporadic cases clinically not suggestive of *ORF15*. We also show how easy it is to analyse female carriers or affected women. We expect that more heterozygous women with severe disease and no family history will be identified by this technique in the future.

**Supplementary Information:**

The online version contains supplementary material available at 10.1007/s00439-025-02807-0.

## Introduction

*RPGR (Retinitis Pigmentosa GTPase Regulator*) is a 172 kb gene located on the X chromosome in the Xp21.1 region. It contains 19 exons and encodes around twenty transcripts with the main transcript, *RPGRex1-19*, expressed in many tissues (lung, kidney, brain, vas deferens) in the transition zone of motile and primary cilia. The retina specific transcript *RPGRorf15* consists of 15 exons, including the terminal exon known as open reading frame (ORF) 15. It encodes a protein of 1152 amino acids (127 kDa), 567 of which are specifically encoded by *ORF15*, a highly repeated region rich in glycines and glutamic acids, representing a mutational hotspot.

Variants in *RPGR* are linked with various inherited retinal degenerations (IRD). They account for more than 70% of X-linked retinitis pigmentosa (XLRP) (Sharon et al. 2000) and for 8% to 28% of sporadic RP (Breuer et al. 2002; Pelletier et al. 2007; Branham et al. 2012). They are associated with severe phenotypes in men, beginning in the first or second decade of life and leading to legal blindness as early as 30–40 years of age. The first symptoms are generally night blindness and peripheral visual field abnormalities. Carrier women can be asymptomatic but usually have fundus abnormalities known as tapetal-like reflex. In some cases, retinal degeneration occurs, but it is often less severe than in men. More than 500 deleterious variants have been identified in *RPGRex1*-19 and *RPGRorf15*. Pathogenic variants found in the *ORF15* are responsible for 60% of IRD caused by *RGPR* (Vervoort et al. 2000). They are almost exclusively truncating variants. They give rise to several clinical presentations such as rod-cone dystrophies (RCD, 70%), cone-rod dystrophies (CRD, 6–23%) or cone dystrophies (CD, 7%). These disorders are characterized by considerable clinical heterogeneity, even in relatives carrying the same variant. The more the variants are located towards the 3’ end of *ORF15*, the more they are linked with phenotypes predominantly affecting cones (Nassisi et al. 2022).

*ORF15* is a repeated region of almost 2 kb, and its molecular analysis using high-throughput sequencing techniques is complicated. Short-read Next Generation Sequencing (NGS) techniques give poor depth of coverage of the region due to misalignment of these short-sequenced DNA fragments. Third-generation techniques, which generate long reads, appear to be best suited to the analysis of repeated regions. Yahya et al. (Yahya et al. 2023) obtained promising results by sequencing *ORF15* with Nanopore (MinION, Oxford Nanopore Technologies) in 54 individuals suffering from retinitis pigmentosa or macular dystrophy. In the era of emerging gene therapies, and given the prevalence of *RPGRorf15* variants, it is essential to develop a technique allowing the molecular diagnosis. Using Nanopore sequencing, we analyzed a cohort of male and female patients with retinal dystrophy for whom short-read sequencing was inconclusive, in order to evaluate the interest and place of this technique in the IRD diagnostic strategy.

## Materials and methods

### Patient recruitment

Patients were diagnosed with RCD, CRD and CD in centres participating in this study (Lille, Montpellier, Rennes University Hospitals). Their DNA samples were collected for molecular diagnosis and retrieved for the study from DNA collection (Base-OPH approval number CODECOH DC-2024-6223). The use of data from Base-OPH has been approved by the CNIL under the number CNIL DR-2023-061. The study protocol adhered to the tenets of the Declaration of Helsinki. Informed consent was obtained for each patient. Twenty-six controls were analysed to validate the technique (Supplementary Table 1), including 16 men and four women, hemizygous or heterozygous carriers for an *RPGRorf15* variant previously identified by Sanger sequencing, as well as six negative controls (five men and one woman).

**Fig. 1:**
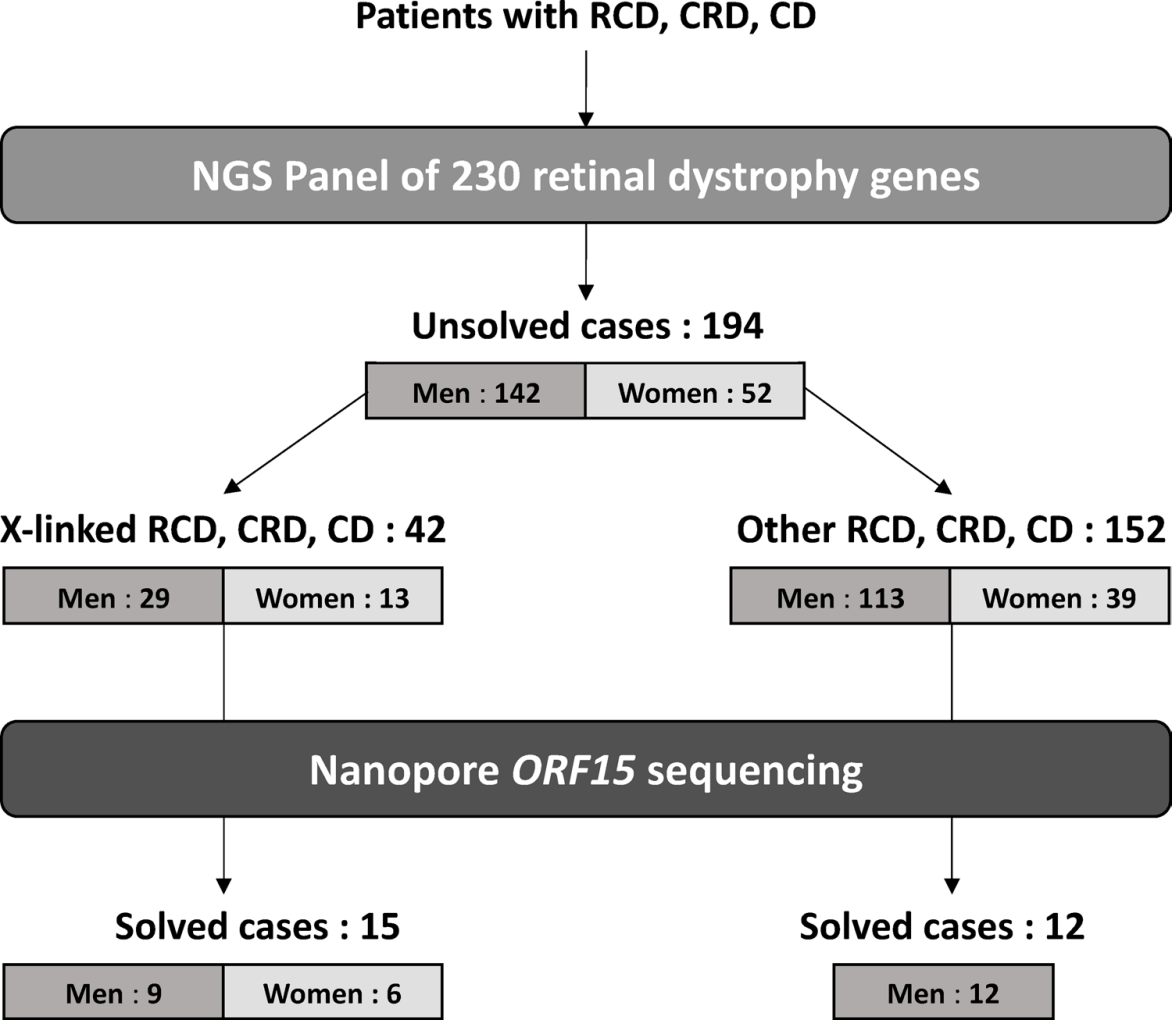
Experimental workflow and phenotype of the patient's cohort. RCD: rod-cone dystrophy, CRD: cone-rod dystrophy, CD: cone dystrophy. Patients with RCD, CRD or CD firstly analysed by a NGS panel of 230 retinal dystrophy genes, with no causal variant identified have been selected for long-read Nanopore sequencing. 194 samples underwent Nanopore sequencing of *RPGR ORF15*. 42 patients were referred for a suspected X-linked retinal dystrophy whereas 152 patients had a different suspected mode of transmission. Among the 152 patients with a suspected autosomal inheritance, 82 males on 113 (72.5%) and 23 females on 39 (58.9%) had no family history or other affected family members. Identification of a disease-causing variant occurs in 27 cases

We then included 194 patients (142 men and 52 women) referred for RCD, CRD or CD with suspected X-linked or autosomal inheritance, genetically unsolved after initial high-throughput sequencing of a panel of 230 IRD genes (Fig. 1). The inclusion criteria for patients were early and/or severe disease in symptomatic men and women. The XLRP genes (*RP2* and *RPGR*) were included in this panel (the complete gene list and technical details are presented in Supplementary Materials). It should be noted that the initial short-read sequencing of *ORF15* provided sufficient quality (> 30X) in most patients in the regions between c.1754 and c.2110 and from c.3390 to c.3459. However, no sequencing was possible in the region from c.2111 to c.3389, which consists of G and A repeats, making bioinformatic alignment unfeasible.

In addition to this initial cohort, we also selected 26 female relatives who were either relatives of *RPGRorf15* variants carriers previously identified by Sanger sequencing or relatives of male patients included in the cohort. These women were either symptomatic, or presented carrier signs (tapetal-like reflex) on fundus examination or were asymptomatic with a normal fundus. For four of them, apart from the family link, no detailed clinical information was available. They are reported with ‘unknown status’.

### Clinical examination

Clinical data were retrospectively collected from medical records. These included sex, age at the time of diagnosis and examination, personal and familial history, visual complaints, best-corrected visual acuity (BCVA) assessed by the Early Treatment Diabetic Retinopathy Study (ETDRS) chart, refractive error, slit-lamp biomicroscopy, static and kinetic visual fields (VFs), full-field electroretinogram (ffERG) using MonColor^®^ unit (Métrovision, Perenchies, France), spectral domain optical coherence tomography (SD-OCT) using Spectralis OCT (Heidelberg Engineering, Inc., Heidelberg, Germany), fundus photography, short wavelength autofluorescence (SWAF) and near-infrared reflectance (NIR) fundus imaging using Heidelberg Retinal Tomograph (Heidelberg Engineering, Inc., Heidelberg, Germany).

### DNA extraction and PCR amplification

DNAs were extracted from patients’ whole blood collected in EDTA tubes. Extraction was automated and performed using the ChemagicStar™ robot (Hamilton^®^, Reno, NV, U.S.A) with DNA blood Chemagic 1 K/240 prep kit (Revvity).

We chose to amplify 2.1 kb amplicons comprising the entire *ORF15* as the basis of the library. Amplicons were obtained from a DNA template normalized to 100 ng/µL per sample. We used *ORF15*-specific primers (E15Flr AGCCAGACAGTTACATGGAAGGTGCAA and E15Rlr TGTCTTTGGCTCCTTAACACAGCTGCATCAG). The PCR mix consisted of 1 µL of genomic DNA, 2.5 µL of 5 mM dNTPs (Eurogentec, Seraing, Belgium), 0.25 µL of Advantage GC genomic LA Polymerase (TaKaRa Bio, Kusatsu, Japan), 12.85 µL of GC Melt Reagent (TaKaRa Bio), 0.5 µL of sense primer (5 pmol/µL) and antisense primer (5 pmol/µL), 7.75 µL of nuclease-free water. Thermal cycler programming (Eppendorf^®^ Mastercycler^®^ Nexus X1, Hamburg, Germany) included a denaturation step at 94 °C for 5 min, followed by 30 cycles of 94 °C for 1 min, 65 °C for 1 min, and 72 °C for 2 min, then a terminal elongation step at 72 °C for 10 min. PCR products were then purified using AMPure XP beads (Beckman Coulter, Brea, CA, USA) and quantified by TapeStation with Genomic DNA kit (Agilent^®^, Santa Clara, CA, USA)(see Supplementary Materials).

### Library preparation

Amplicons were prepared with the NEBNext ultra II End repair/dA Tailling module (New England Biolabs^®^, Evry-Courcouronnes, France). After a washing and elution step, they were quantified with the Qubit™ dsDNA HS Assay kit (Invitrogen™, Waltham, MA, USA). They were then barcoded using the Native Barcoding Kit 24 V14 (Oxford Nanopore Technologies, Oxford, UK), enabling up to 24 patients to be mixed. The library was prepared according to the supplier’s protocol.

### Long-read sequencing and sequence analysis

All 194 DNA samples underwent long-read sequencing using the Oxford Nanopore Technologies (ONT). The library (20 fmol) was loaded onto a FLO-PRO114M flow cell (up to 2,273 pores, ONT) with 500 µL of priming mix after a wash step with Flow Cell Wash Kit (ONT) as long as the number of available pores remains greater than or equal to 500 nanopores. A 2 to 3 h run was then launched on PromethION 2 (ONT). Basecalling and conversion of POD5 files to FastQ were performed with MinKNOW 7.2.14 in SUP mode and mapped on GRCh38 with minimap2 2.24. Each patient’s BAM files were analysed manually with IGV v2.17.0 (Integrative Genomics Viewer) and Alamut™ Visual Plus (Sophia Genetics™, Switzerland). The samples were sequenced by run of 24. Note that we used flow cells previously used for another long-read sequencing project.

The search for variants was carried out by uploading BAM files on IGV (https://igv.org/) and Alamut™ Visual Plus software version 1.5.1 (https://www.sophiagenetics.com/). Annotation was performed according to HGVS guidelines (https://hgvs-nomenclature.org/). The variations highlighted were classified according to the American College of Medical Genetics (ACMG) classification (Richards et al. 2015).

### Sanger sequencing

All variants found by long-read sequencing were controlled by Sanger sequencing. In addition, family segregation was performed for some relatives of c.2719G > T variant carriers. Primers and conditions are available in Supplementary Materials.

## Results

### Cohort description

Among the 194 patients sequenced by long-read sequencing (LRS), 42 were referred specifically for X-linked retinal dystrophy and 152 had sporadic (*n* = 105) or suspected autosomal (*n* = 47) RCD, CRD or CD (Fig. 1). The entire cohort counted 142 men and 52 women (Table 1). Age in the cohort followed a normal distribution (D’Agostino & Pearson test, *p* = 0.2676). Of the 194 patients, only four were related.

**Table 1:** Reported phenotype of patients in the cohort

Reason for patient referral	No./Total No. (%)
**X-linked rod-cone dystrophy (XL-RCD)**	
Male	29/142 (20.4%)
Female	13/52 (25%)
Symptomatic	11/13
Unknown status	2/13
**Autosomal or sporadic rod-cone dystrophy (RCD)**	
Male	73/142 (51.4%)
Autosomal inheritance suspected	19/73
Sporadic	54/73
Female	22/52 (42.3%)
Autosomal inheritance suspected	9/22
Sporadic	13/22
**Autosomal or sporadic cone-rod dystrophy (CRD)**	
Male	18/142 (12.7%)
Autosomal inheritance suspected	4/18
Sporadic	14/18
Female	12/52 (23.1%)
Autosomal inheritance suspected	4/12
Sporadic	8/12
**Autosomal or sporadic cone dystrophy (CD)**	
Male	18/142 (12.7%)
Autosomal inheritance suspected	5/18
Sporadic	13/18
Female	5/52 (9.6%)
Autosomal inheritance suspected	3/5
Sporadic	2/5
**Male with undetermined retinal dystrophy (RD)**	4/142 (2.8%)
Autosomal inheritance suspected	3/4
Sporadic	1/4

194 patients were included in the cohort, including 142 men and 52 women. The number of male and female patients is shown for each phenotype

In men, 29 were suspected of having X-linked RCD based on pedigree analysis (Table 1), four were referred for IRD but no further clinical information was available, and the others were either considered sporadic or suspected of having autosomal transmission. In the latter group, 73 men were referred for RCD, 18 for CRD and 18 for CD.

Among the 52 women, 13 were suspected of having X-linked RCD based on family history (11 had symptoms of IRD and two had not undergone clinical examination), 23 had been diagnosed with sporadic IRD, and 16 were suspected of having autosomal transmission. In this sporadic or autosomal group, 22 women had RCD, 12 had CRD, and five had CD (Table 1).

### Long-read sequencing metrics

We first validated the technique on two initial runs of respectively 12 controls (10 positives and 2 negatives) and 14 controls (10 positives and 4 negatives). In the first run, Nanopore sequencing with PromethION (ONT) was able to generate 0.7 Gb of data and 377 kb of reads with a mean length of 2 kb in two hours of run, corresponding to the full-length *ORF15* sequence. The mean depth was 20,673reads (σ = 1,532) per sample with a ≥ 30 X coverage of 100% for each sample (Table 2). The pathogenic variants of the positive controls were correctly called and aligned. No pathogenic variants were found in the negative controls.

**Table 2:** Quality data of long-read Nanopore sequencing

*N*° Run	Number of patients/Run	Mean reads per patient	Standard deviation	Median reads per patient	Max reads per patient	Min reads per patient	Coverage ≥ 30X	Data/Run (Gb)	Run duration	Mean Gb/h
Run 01	12	20,673	1,532	20,670	23,691	18,537	100.00%	0.7	2 h	0.35
Run 02	24	2,013	392	2,116	2,733	1,172	100.00%	2.11	2 h 17 min	0.92
Run 03	24	1,903	599	1,821	3,047	894	100.00%	2.2	2 h 9 min	1.02
Run 04	22	1,004	184	1,037	1,267	567	100.00%	15.01	3 h 1 min	4.74
Run 05	23	1,302	732	1,079	3,693	464	100.00%	2.72	2 h 54 min	0.94
Run 06	24	13,052	31,920	7,039	162,727	2,360	100.00%	13.27	3 h 8 min	4.24
Run 07	24	2,468	668	2,436	1,098	3,936	100.00%	6.07	3 h 19 min	1.83
Run 08	23	3,636	1,486	3,677	538	7,041	100.00%	6.48	2 h 26 min	2.66
Run 09	22	6,567	1,520	7,363	3,979	10,413	100.00%	9.95	2 h 2 min	4.89
Run 10	24	2,084	1,204	1,662	5,592	324	100.00%	4.63	2 h 5 min	2.22
Run 11	24	4,406	2,509	3,783	12,536	927	100.00%	7.08	1 h 58 min	3.6
Mean of all runs	22	5,373	6,137	2,468	20,082	4,240	100.00%	6.38	2h27	2.64

For the patients to be tested (runs 2 to 11), 1 h run generated an average of 2.64 Gb of data (σ = 1.53). The average number of reads per patient per run was 5,373 (σ = 6,137). The main sequencing data are summarised in Table 2.

An average of 1,395 pores (σ = 563.2) were available at the start of the runs (Supplementary Table 2). We also observed a rapid blockage of usable pores, leading to a drop in efficiency. In an attempt to remedy this without a wash step during the run, we programmed “pore scan” steps, i.e. a period during the run when the pores try to eject the blocked fragment to become available again. As the data obtained in this way were sufficient, we did not resort to DNase I treatment. On the first patient run (run 02), these were programmed by default every 1.5 h, enabling us to recover 551 functional pores at 1.5 h out of the 864 usable at the start of the run. However, after 2 h, there were virtually no pores left. To improve run performance, we then set up a pore scan every 30 min, almost doubling the number of reads generated (around 76 kb for run 02 versus 134 kb for run 03). The average number of pores remaining at the last pore scan was 341 pores (σ = 148.2).

### Identification of *ORF15* pathogenic variants

Disease-causing variants were found in 27/194 patients (13.9%). Twenty-one patients were men and six were women (Fig. 1). Of the 42 patients in whom X-linked transmission was suspected, 15 had a pathogenic variant in *ORF15* (35.7%). Nine were men, five of whom had RCD (55.6%), one had CRD (11.1%) and one had CD (11.1%). No clinical information was available for two patients (22.2%). The six women, suspected of XLRP, were all symptomatic, presenting clinically RCD.

Of the 152 patients in whom another mode of inheritance was suspected, only 12 carried pathogenic *ORF15* variants (7.9%). They were all male patients, three presented RCD (25%), five CRD (41.7%), and four CD (33.3%). Of these 12 patients, eight were sporadic cases and four had a suspected autosomal mode of inheritance, two recessive CD and two recessive CRD patients, reporting affected brothers during questioning. Of note, all four patients had a predominantly cone phenotype, less suggestive of *RPGR*-related retinal dystrophies, explaining why clinicians considered other diagnostic hypotheses.

**Fig. 2:**
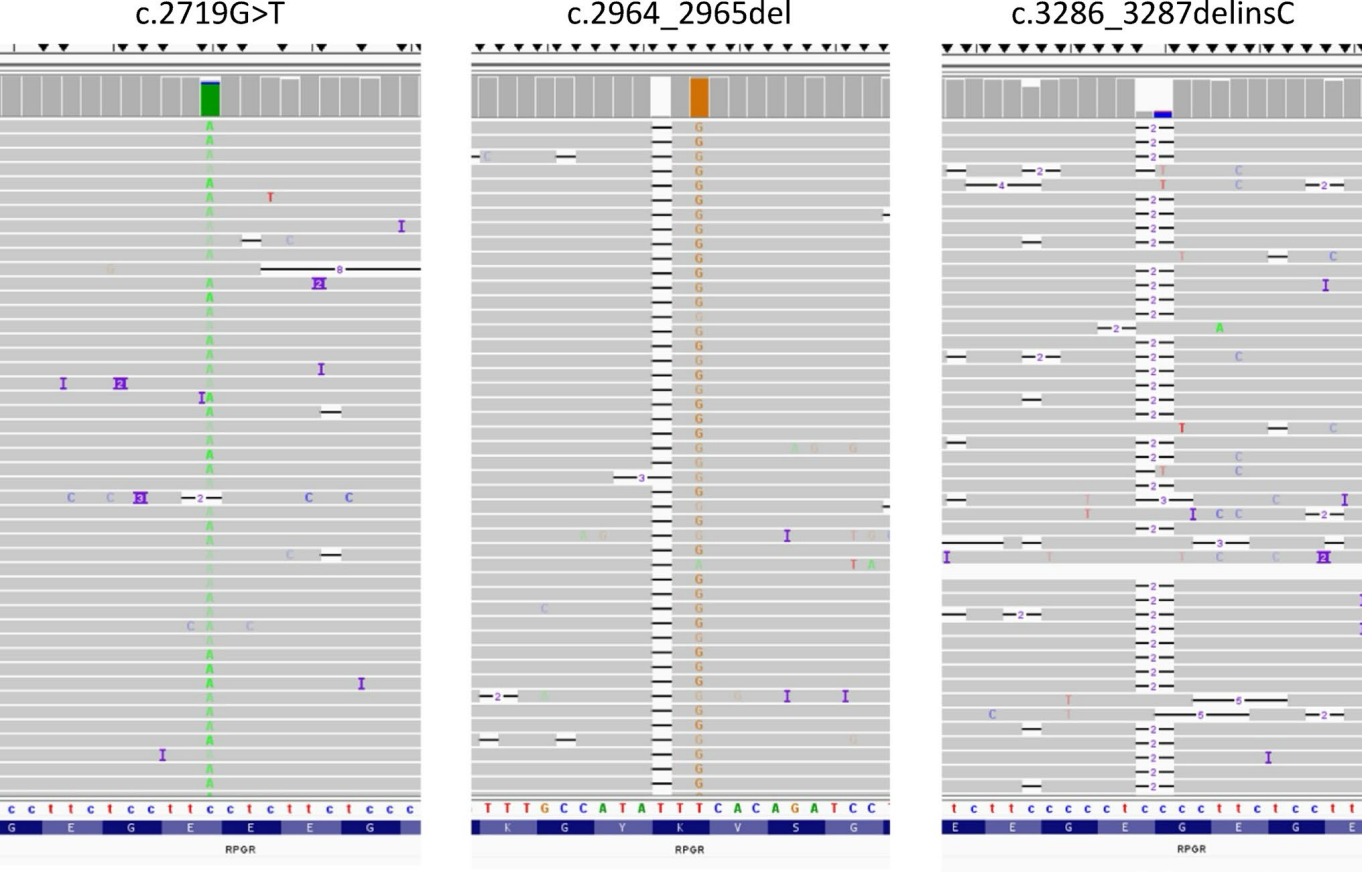
Variant visualization with Integrative Genomics Viewer (IGV). BAM files screenshot showing three different variant types identified by long-read sequencing

Eighteen distinct variants in *ORF15* were identified (Table 3). All were truncating variants (nonsense or frameshift) and classified as pathogenic or likely pathogenic according to the American College of Medical Genetics (ACMG) classification. They were two nucleotide substitutions, 15 small deletions and one insertion (Fig. 2). Four of these variants, namely c.2923_2924insGAAAGGGG, c.3119_3123del, c.3153del, c.3231_3234del, have never been reported to our knowledge. The c.2719G > T variant is the most frequently found, occurring in five men, three of whom presented RCD and two with CRD. Noteworthy, all these patients were from the Hauts-de-France region of France. Three men harbored a c.2236_2237del variant, two with RCD and one with CRD. Most of variants located in the 3’ end part of *ORF15* (after the Glu1000 residue): c.3071_3080del, c.3119_3123del, c.3153del, c.3178_3179del, c.3231_3234del were associated with CRD (*n* = 1) or CD (*n* = 4). Clinical examination was not available for two patients harboring the c.3178_3179del and c.3119_3120del variants. An 8 bp insertion, c.2923_2924insGAAAGGGG was clearly visible on the BAM file but had been incorrectly annotated, necessitating Sanger sequencing for confirmation.

**Table 3:** Description of carriers of *RPGR ORF15* variants

Patient	Sex	Age	Status	Variation	Protein change	Reference
Rod-cone dystrophy						
1	M	26	Hemizygous	c.2719G > T	p.(Glu907*)	Ayyagari et al. 2002
2	M	20	Hemizygous	c.2719G > T	p.(Glu907*)	Ayyagari et al. 2002
3	M	39	Hemizygous	c.2330_2331del	p.(Lys777Argfs*57)	Sheng et al. 2010
4	M	32	Hemizygous	c.2719G > T	p.(Glu907*)	Ayyagari et al. 2002
5	M	36	Hemizygous	c.2405_2406del	p.(Glu802Glyfs*32)	Vervoort et al. 2000
6	M	27	Hemizygous	c.2964_2965del	p.(Glu989Glyfs*89)	Garcia-Hoyos et al. 2006
7	M	12	Hemizygous	c.2236_2237del	p.(Glu746Argfs*23)	Vervoort et al. 2000
8	M	37	Hemizygous	c.2236_2237del	p.(Glu746Argfs*23)	Vervoort et al. 2000
9	F	20	Heterozygous	c.2476_2477del	p.(Arg826Glyfs*8)	Pusch et al. 2002
10	F	39	Heterozygous	c.2894del	p.(Glu965Glyfs*124)	Vervoort et al. 2000
11	F	56	Heterozygous	c.2894del	p.(Glu965Glyfs*124)	Vervoort et al. 2000
12	F	50	Heterozygous	c.2964_2965del	p.(Glu989Glyfs*89)	Garcia-Hoyos et al. 2006
13	F	47	Heterozygous	c.2506del	p.(Glu836Lysfs*253)	Pelletier et al. 2007
14	F	37	Heterozygous	c.2323_2324del	p.(Arg775Glufs*59)	Breuer et al. 2002
Cone-rod dystrophy						
15	M	30	Hemizygous	c.2719G > T	p.(Glu907*)	Ayyagari et al. 2002
16	M	44	Hemizygous	c.2719G > T	p.(Glu907*)	Ayyagari et al. 2002
17	M	48	Hemizygous	c.2218G > T	p.(Glu740*)	Rozet et al. 2002
18	M	37	Hemizygous	c.2236_2237del	p.(Glu746Argfs*23)	Vervoort et al. 2000
19	M	34	Hemizygous	c.3231_3234del	p.(Asn1077Lysfs*11)	This study
20	M	61	Hemizygous	c.2840del	p.(Glu947Glyfs*142)	Pelletier et al. 2007
Cone dystrophy						
21	M	7	Hemizygous	c.2923_2924insGAAAGGGG	p.(Glu975Glyfs*117)	This study
22	M	33	Hemizygous	c.3178_3179del	p.(Glu1060Argfs*8)	Garcia-Hoyos et al. 2006
23	M	28	Hemizygous	c.3119_3123del	p.(Glu1040Glyfs*37)	This study
24	M	32	Hemizygous	c.3071_3080del	p.(Glu1024Glyfs*62)	Nassisi et al. 2022
25	M	52	Hemizygous	c.3153del	p.(Gly1052Glufs*37)	This study
X-linked retinal dystrophy without phenotypic precision						
26	M	73	Hemizygous	c.3178_3179del	p.(Glu1060Argfs*8)	Garcia-Hoyos et al. 2006
27	M	82	Hemizygous	c.3119_3120del	p.(Glu1040Glyfs*38)	Nassisi et al. 2022

Disease-causing variants were found by long-read sequencing in 27 samples: 14 have rod-cone dystrophy, six present with cone-rod dystrophy, five with cone dystrophy and clinical data were not available for two of them. Four variations have never been described in the literature

Benign variants were also found, of all types: nucleotide substitutions, small deletions and duplications, insertions, and most of them segregated in complex allele (Supplementary Table 3).

### Related female carriers of *ORF15* variants analysed by long-read sequencing

Long-read sequencing was also used to analyse 26 female relatives of male patients from the initial cohort as well as previously diagnosed male index cases from the laboratory (Table 4). A targeted LRS was therefore performed, allowing us to identify *ORF15* variants in 10 females (Supplementary Table 4), all of them with a family history of XLRD (10/26, 38.5%). Two of them presented RCD and four others had tapetal-like reflex. The clinical status of four women was unknown. It should be noted that there are no de novo cases. Whenever the mothers of patients carrying *ORF15* variants were sequenced, they were heterozygous for their son’s variant. All 14 female relatives of male sporadic patients from the cohort and for whom no variant was found, were asymptomatic and as expected harbored no *ORF15* variant.

**Table 4:** Reported phenotype of sequenced female relatives. 26 female relatives were sequenced by long-read Nanopore Sequencing

**Relatives’ phenotype: No./Total No. (%)**	Relatives of X-linked RCD, CD, CRD patients (probands sequenced by Sanger method) 12/26 (46.2%)	Relatives of non X-linked RCD, CD, CRD patients (probands sequenced by LRS) 14/26 (53.8%)
**Clinical involvement**	Rod-cone dystrophy 2/12 Carrier’s sign (tapetal reflects) 4/12 Asymptomatic 2/12 Unknown Status 4/12	Asymptomatic 14/14
**Number of positive cases**	10/12	0/14

Twelve subjects were relatives of *ORF15* variant carriers previously identified by Sanger sequencing, among them ten also carried an *ORF15* variant. Fourteen were asymptomatic relatives of male patients sequenced by Nanopore sequencing in the cohort, but without any *ORF15* variant identified. As expected none of the fourteen women had any variant

### Phenotype in a family carrying the c.2719G > T variant

**Fig. 3:**
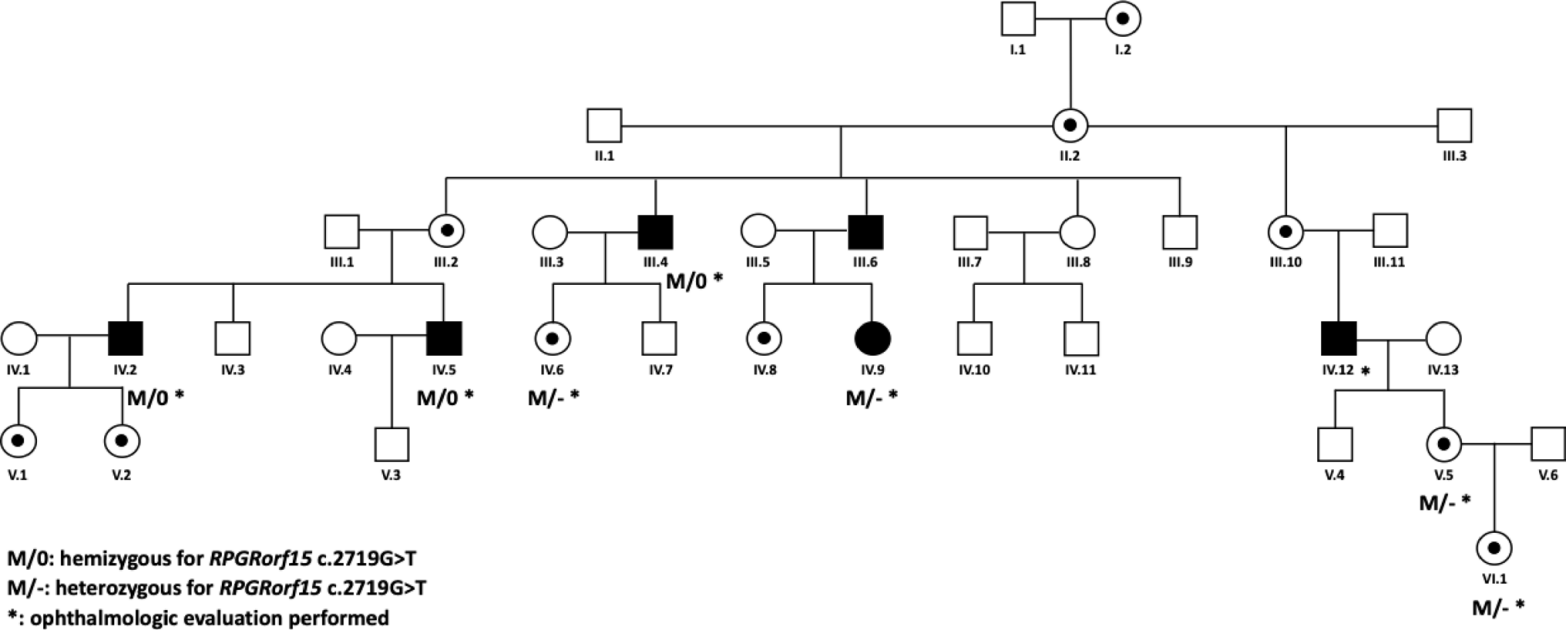
Family tree of the studied c.2719G>T variant carriers’ family. Five men present with XLRP, four of them have cone-rod dystrophy (III.4, IV.2, IV.5 and IV.12). One woman was symptomatic with CRD in one eye (IV.9), Three women (IV.6, V.5 and VI.1) were asymptomatic but presented carrier signs (tapetal-like reflex). DNA samples were available for seven family members (III.4, IV.2, IV.5, IV.6, IV.9, V.5 and VI.1) and all received molecular diagnosis

**Fig. 4:**
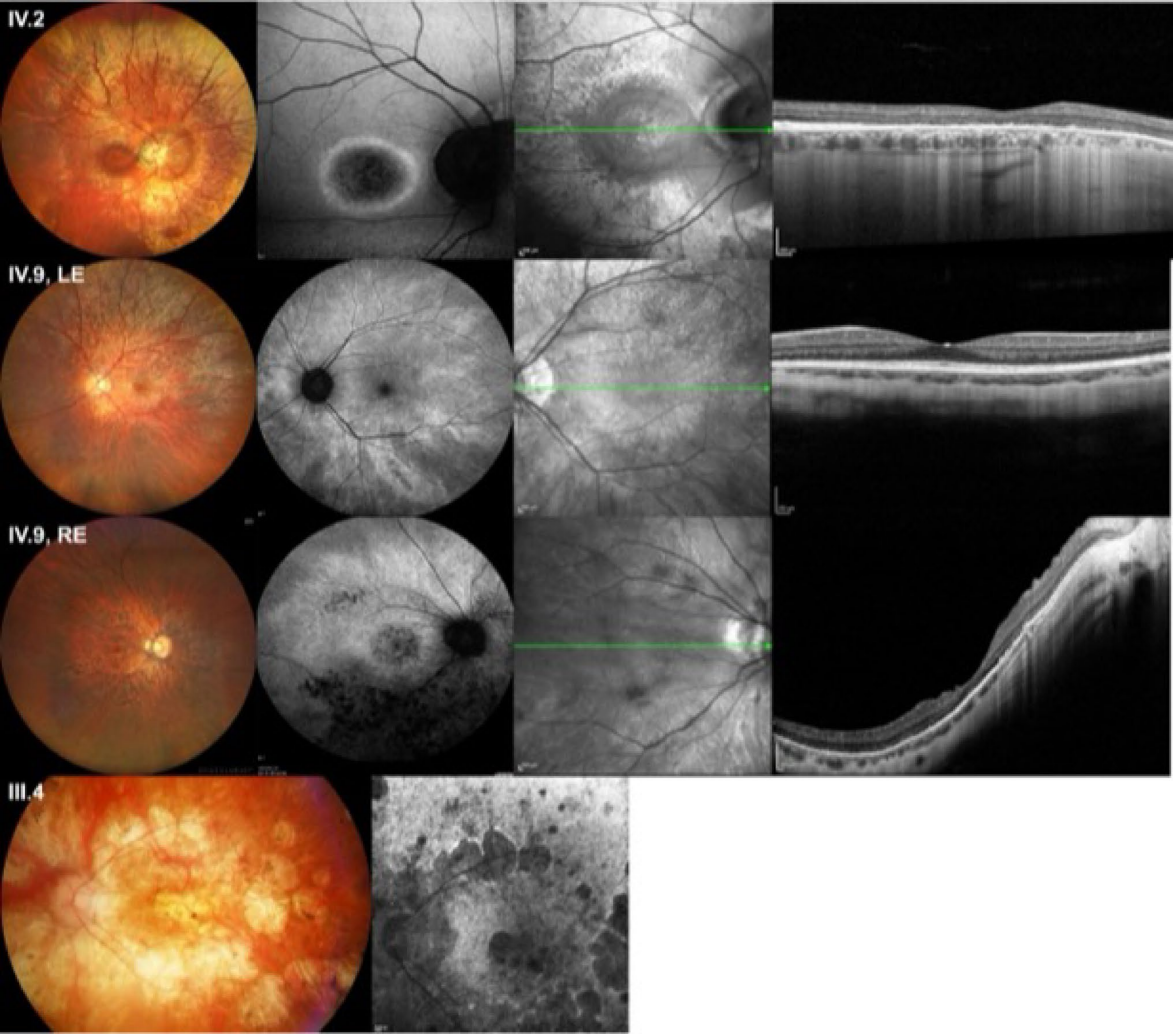
Clinical examination of patients IV.2, IV.9 and III.4. Patient IV.2 (top row): bull's-eye maculopathy with hypoautofluorescent macular lesion on SWAF. Patient IV.9 left eye (second row): tapetal-like reflex. Patient IV.9 right eye (third row): fundus shows macular and chorioretinal atrophy. Round foveal and midperipheral autofluorescence on SWAF. On HD-OCT, disappearance of outer retinal layers. Note the difference between right and left eyes. Patient III.4 (bottom row): advanced CRD. Patchy chorio-retinal atrophy on SWAF, round patches of macular and midperipheral hypoautofluorescence

The c.2719G > T variant has already been reported by Nassisi et al., (Nassisi et al. 2022) in one patient with cone-rod dystrophy. In our study, it was identified by Nanopore sequencing in five unrelated male patients, all referred by the “Exploration de la Vision et Neuro-Ophtalmologie” Department of the Lille University Hospital. To explore a possible founder effect and to characterize the phenotype associated with this variation, we looked for other carriers of this variant identified in our laboratory by Sanger sequencing. We found 13 other patients, eight men and five women, belonging to three different families, all from the same geographical area of France. Detailed clinical information was available for eight affected members of one family (Figs. 3 and 4). Among these patients, four men presented CRD (III.4, IV.2, IV.5 and IV.12). Patient IV.2 (Fig. 4, top row) at the age of 44 presented a bull’s-eye maculopathy with a persistent tapetal-like reflex on fundus examination. A round hypoautofluorescent macular lesion bordered by a hyperautofluorescent ring was observed on SWAF. It corresponded to a foveal loss of outer retinal layers at HD-OCT. Patient III.4 at the age of 55 (Fig. 4, bottom row) presented a more advanced retinal degeneration with large areas of chorioretinal atrophy in the posterior pole and severely impaired scotopic and photopic ERG responses, although initially the pattern was of CRD. Patient IV.9 was a 29 y.o. woman with a very asymmetrical condition, with BCVA 20/100 in the right eye and 20/20 in the left eye. On fundus examination, the right eye showed macular involvement close to her cousin IV.2, while the examination of the left eye revealed only tapetal-like reflex typical of carrier women. (Fig. 4, middle rows). Three women (IV.6, V.5 and VI.1), were asymptomatic but presented carrier signs (tapetal-like reflex) on the fundus examination. Their BCVA and ERG were normal. One man was reported as affected in the pedigree but no further clinical information was available (III.6).

## Discussion

### Contribution of long-read Nanopore sequencing to the study of *ORF15*

Our study confirms long-read Nanopore sequencing as a promising approach for the analysis of *ORF15* to study a large cohort of IRD patients unsolved after a first NGS of short-reads. We analysed 142 male and 52 female patients with RCD, CRD, or CD and identified truncating variants in 27 patients (13.9%), 21 men and 6 women. The coverage and depth data for these variants were very satisfactory, and enabled the identification of *ORF15* hemizygous but more importantly heterozygous variants more quickly and efficiently than with Sanger sequencing. As the region is relatively small, the amplicon sequencing strategy enabled high depth and accurate detection of single-nucleotide variants, rendering adaptive sampling unnecessary. This significant percentage of resolved cases confirms the interest of including *ORF15* long-read sequencing in routine strategies, particularly in patients with early retinal damage in whom first-line explorations were negative, even without clinical and family suspicion of X-linked transmission. Notably, patients with cone involvement were over-represented in sporadic cases in which a *ORF15* variant was found (5/8 subjects), suggesting the value of studying *ORF15* by long-read sequencing in second-line in unsolved cases of CRD/CD. In another cohort of simplex male patients with CRD, variants in *ORF15* were found in 13.8% of cases (Branham et al. 2012), which led the authors to propose that *RPGRorf15* should instead be considered as a first-tier gene for screening in sporadic CRD in men.

We observed a lower frequency of *ORF15* variants among patients with XLRP (9 out of 29 male patients, 31%) compared to the literature (Vervoort et al. 2000). This difference is explained by a selection bias in our study. The patients were selected from those in whom no pathogenic variant was found with the 230 gene panel performed in our laboratory. The vast majority of patients referred for XLRP was pre-screened for *ORF15* as first-line testing using the direct Sanger sequencing. Only XLRP patients negative for *RP2* or *RPGR1-19* variants after NGS panel and patients in whom the mode of inheritance was uncertain have been included in this study, explaining the low proportion of XLRP patients in our cohort. Concerning isolated RCD/CRD, the frequency of patients with *ORF15* variant varies according to studies (Breuer et al. 2002; Pelletier et al. 2007; Branham et al. 2012). In our work, we found *ORF15* variations in 7.5% of sporadic cases, compared to 24% in another cohort of French patients (Pelletier et al. 2007). Here again, a selection bias could be at the origin of these differences, the clinical criteria for inclusion of patients being less evocative of an X-linked transmission in our study than in that of Pelletier et al., (Pelletier et al. 2007): early and/or severe photoreceptor damage only. In addition, our cohort presented a higher number of sporadic IRDs (*n* = 107 versus *n* = 25). In conclusion, the diagnostic yield of XLRP depends on the lab strategy: if all men with IRD are pre-screened by Sanger for *ORF15*, as in some studies (Nassisi et al. 2022), the variant detection rate is higher than in our lab, in which we use the NGS IRD panel as a first-line test with its inherent biases (low coverage and difficulty of reads alignment of *ORF15*).

### Technical limitations

We observed no failures in variant identification by LRS, but we did detect errors in the annotation, as with the 8 bp insertion c.2923_2924insGAAAGGGG identified by LRS, for which a Sanger sequencing was necessary to confirm the annotation. Other technical limitations have already been reported by Vaché et al., (Vaché et al. 2025) for variants found in a highly repeated region of *ORF15* (c.2506del, c.2792del and c.2931_2932insAAAGG) described as refractory to sequencing due to high error rates and alignment difficulties. It should be noted that we did not encounter the same issue with the c.2506del variant also identified in our cohort. We also hypothesized an effect of the variant type. However, duplication (c.2283_2311dup) and delins (c.3286_3287delins) sequenced as controls were correctly identified with Nanopore sequencing, while they were located outside this highly adenine and guanine-rich zone. Sanger sequencing may still be required for variants located in this particular area of the *ORF15* region to ensure correct annotation. Patients’ data were manually analysed by reviewing BAM files on IGV and Alamut. Ultimately, we aim to develop bioinformatics pipelines to facilitate variant reading and annotation. However, the challenges described above may make this development tricky.

We also encountered the problem of a reduction in the number of pores available for sequencing during runs, with a significant drop in the first 30 min. By changing the pore scan settings to once every half hour, we were able to obtain a sufficient amount of data (on average of 2.40 GB/h) in 2 to 3 h of running, without the need for a wash step.

### The 3’ end of *ORF15* is more associated with cone-rod dystrophies

Analysis of the 27 patients carrying a pathogenic variation in the *ORF15* revealed a predominance of the RCD phenotype in both males and females. However, for the c.2719G > T and the c.2236_2237del variants, identified in respectively five and three unrelated patients in our cohort, a phenotypic variability was noted as both variants were associated with RCD (*n* = 3 and *n* = 2 respectively) as well as CRD (*n* = 2 and *n* = 1 respectively). Furthermore, the ‘watershed zone’ initially described between residues 949 and 1047 by De Silva et al., (De Silva et al. 2021) can, in our cohort, be extended to the entire *ORF15* with the exception of the part coding for the C-terminal basic domain. Indeed, from amino acid 1024, we observed a clear predominance of cone damage without any case of RCD. These results are consistent with those of previous cohort studies in *ORF15* (Cehajic-Kapetanovic et al. 2020; Nassisi et al. 2022).

## Phenotype-genotype correlation of the c.2719G > T variant

We took a closer look at the c.2719G > T variant found in five unrelated male patients from our cohort as their families all dwelt in the Hauts-de-France region of France, fact suggestive of a founder effect. Thirteen other carriers of this variation were found in the database of our laboratory, from three distinct families. As detailed clinical information was available for eight members of one family, we focused on the phenotype associated with this variant. The clinical examination of these patients revealed a homogenous phenotype of more or less severe X-linked CRD. In the other families, patients were reported to have an RP with no additional information about the exact IRD subtype (RCD/CRD/CD). We hypothesized a founder effect, but the small size of the population limits the study of this trend. A search for a common haplotype in these patients would be necessary to support this hypothesis.

## Long-read sequencing as a suitable method for analysing women

Few cohorts of women have been described in the literature, certainly because of the technical difficulties in diagnosis. The largest study is that of Fahim et al. (Fahim et al. 2020) who assessed skewed X-chromosome inactivation to explain the clinical severity in 77 women from 41 families, carrying variants in *RPGR*, 39 of which were located in *ORF15*. Of these 77 women, 9 were asymptomatic, 37 were mildly affected, 18 were moderately affected, and 13 were severely affected. This highlights the fact that *ORF15* analysis concerns both female carriers related to an affected male, with highly variable clinical pictures, as in the case of the family presented, but also sporadic cases with RCD or CRD, when no male is affected. In this case, it is essential to carry out a precise ophthalmic examination to look for the characteristic signs of X-linked forms, namely a starburst tapetal-like reflex. In our cohort, women with these fundus signs were directly targeted for *ORF15* sequencing, and no NGS panel was performed, which explains why fewer women were included in the study than men. Twenty-six women related to positive index cases or to male patients in the cohort were also tested by LRS, which underlines the interest of this technique for the study of relatives, instead of Sanger sequencing.

## Conclusion

Long-read approach using PromethION2 (Oxford Nanopore Technologies) allows efficientand accurate detection of *ORF15* variants in repeated regions, providing a precise diagnosis not only for patients with XLRP, but also for those with IRD for which short-read sequencing analysis failed to identify a causal variant. This technology makes it possible to analyse both men and women, for whom analysis of this complex region was often not possible. It is expected that more sporadic female probands will now be discovered. This approach is therefore very promising and could soon be used in routine diagnostic strategies as a first-line test for patients suspected of having X-linked IRD and as a second-line test for unsolved cases.

## Supplementary Information

Below is the link to the electronic supplementary material.

Supplementary Material 1

## Data Availability

The data that support this study are available from the corresponding author upon reasonable request.
