# Supplementary material for: RPGRorf15 nanopore long-read sequencing improves retinitis pigmentosa molecular diagnosis for men and women
Source: Hum Genet. 2026 Feb 13;145(1):20. doi: 10.1007/s00439-025-02807-0 (PMC12904915; doi:10.1007/s00439-025-02807-0)
Supplement: Supplementary file 1 — Supplementary Material 1 [file 439_2025_2807_MOESM1_ESM.docx]

**Supplementary material**

**RPGRorf15 Nanopore long-read sequencing improves retinitis pigmentosa molecular diagnosis for men and women**

Manon Fabard^1^, Aurore Devos^1^, Anaïs F. Poncet^1^, Jean-Pascal Meneboo^2^, Martin Figeac^2^, Céline Villenet^2^, Isabelle Drumare^3^, Sabine Defoort-Dhellemmes^3^, Isabelle Meunier^4,5^, Xavier Zanlonghi^6^, Olivier Grunewald^1^, Vincent Huin^1^, Claire Lecigne^1^, Vasily Smirnov^1,3^, Claire-Marie Dhaenens^1#^

**Supplementary Table 1** List of 26 controls sequenced by long-read Nanopore sequencing

| **N°** | **Sex** | **Status** | **Variation** | **Sequencing Run** |
| --- | --- | --- | --- | --- |
| 1 | M | Positive | c.2719G>T | Run 01 |
| 2 | M | Positive | c.2455_2468del | Run 01 |
| 3 | M | Negative | - | Run 01 |
| 4 | M | Positive | c.2792del | Run 01 |
| 5 | M | Positive | c.3286_3287delins | Run 01 |
| 6 | M | Positive | c.2405_2406del | Run 01 |
| 7 | M | Positive | c.2468_2472del | Run 01 |
| 8 | M | Positive | c.3119_3123del | Run 01 |
| 9 | M | Positive | c.3160G>T | Run 01 |
| 10 | M | Negative | - | Run 01 |
| 11 | M | Positive | c.2236_2237del | Run 01 |
| 12 | M | Positive | c.2488_2494dup | Run 01 |
| 13 | M | Negative | - | Run 02 |
| 14 | M | Negative | - | Run 02 |
| 15 | F | Positive | c.2719G>T | Run 02 |
| 16 | F | Positive | c.3178_3179del | Run 02 |
| 17 | M | Positive | c.2937_2938del | Run 02 |
| 18 | F | Positive | c.2442_2445del | Run 02 |
| 19 | M | Negative | - | Run 02 |
| 20 | F | Positive | c.2683G>T | Run 02 |
| 21 | M | Positive | c.2683G>T | Run 02 |
| 22 | M | Positive | c.3286_3287delins | Run 02 |
| 23 | M | Positive | c.2468_2472del | Run 02 |
| 24 | M | Positive | c.2792del | Run 02 |
| 25 | F | Negative | - | Run 02 |
| 26 | M | Positive | c.2283_2311dup | Run 02 |

**Supplementary Table 2** Numbers of pores available for sequencing at each pore scan time (min) for each run of tested patients. For Run 02 (controls and patients’ samples), pore scan steps were programmed every 1.5 hours. The following runs (only patient’ samples) had a pore scan step every thirty minutes

| **Pore scan time (min)** | **Run 02** | **Run 03** | **Run 04** | **Run 05** | **Run 06** | **Run 07** | **Run 08** | **Run 09** | **Run 10** | **Run 11** |
| --- | --- | --- | --- | --- | --- | --- | --- | --- | --- | --- |
| Start | 864 | 1,129 | 745 | 609 | 1,797 | 1,273 | 1,694 | 2,273 | 1,539 | 2,031 |
| 30 | - | 801 | 689 | 479 | 1,743 | 1,069 | 1,596 | 1,282 | 990 | 1,117 |
| 60 | - | 686 | 617 | 359 | 769 | 702 | 561 | 531 | 679 | 662 |
| 90 | 551 | 464 | 543 | 127 | 401 | 338 | 220 | 262 | 437 | 333 |
| 120 | - | STOP | 532 | 115 | 331 | 227 | 297 | STOP | STOP | STOP |
| 150 | STOP |  | 524 | 102 | 248 | 194 | STOP |  |  |  |

**Supplementary Table 3** SNPs identified by long-read Nanopore sequencing. Some SNPs were found to always segregate together, their association is indicated

| **Variation** | **Protein Change** | **ACMG Classification** | **Segregation** |
| --- | --- | --- | --- |
| c.1754-103C>T | p.(?) | Benign | c.2223G>A and c.2541_2561del |
| c.2223G>A | p.(Glu741=) | Benign | c.1754-103C>T and c.2541_2561del |
| c.2341G>A | p.(Ala781Thr) | Benign | c.2667_2669del |
| c.2541_2561del | p.(Glu850_Gly856del) | Benign | c.1754-103C>T and c.2223G>A |
| c.2606_2620del | p.(Glu869_Glu873del) | Likely Benign | c.*72A>G and c.3231T>A |
| c.2667_2669del | p.(Glu890del) | Benign | c.2341G>A |
| c.2841_2842insTCCCCTTCTCCATCCTCCCCT | p.(?) | Likely Benign | c.3396C>T |
| c.2898C>A | p.(Gly966=) | Benign |  |
| c.2976_2990del | p.(Glu994_Glu998del) | Likely Benign |  |
| c.3060_3071del | p.(Val1025_Glu1028del) | Benign |  |
| c.3074_3085del | p.(Val1025_Glu1028del) | Benign |  |
| c.3219C>T | p.(Gly1073=) | Benign |  |
| c.3231T>A | p.(Asn1077Lys) | Benign | c.*72A>G and c.2606_2620del |
| c.3264G>A | p.(Val1088=) | Benign |  |
| c.3396C>T | p.(Asn1132=) | Benign | c.2841_2842insTCCCCTTCTCCATCCTCCCCT |
| c.3430G>A | p.(Val1144Ile) | Benign |  |
| c.*72A>G | p.(?) | Likely Benign | c.2606_2620del and c.3231T>A |

**Supplementary Table 4** Description of the 10 female relatives carrying a *ORF15* variants

| **Patient** | **Sex** | **Age** | **Status** | **Variation** | **Protein change** | **Reference** |
| --- | --- | --- | --- | --- | --- | --- |
| **Rod-cone dystrophy** | | | | | | |
| 1 | F | 24 | Heterozygous | c.2236_2237del | p.(Glu746Argfs*23) | Vervoort et al. 2000 |
| 2 | F | 13 | Heterozygous | c.2683G>T | p.(Gly895*) | Pelletier et al. 2007 |
| **Carriers’ signs** | | | | | | |
| 3 | F | 17 | Heterozygous | c.2683G>T | p.(Gly895*) | Pelletier et al. 2007 |
| 4 | F | 19 | Heterozygous | c.2683G>T | p.(Gly895*) | Pelletier et al. 2007 |
| 5 | F | 33 | Heterozygous | c.2719G>T | p.(Glu907*) | Ayyagari et al. 2002 |
| 6 | F | 14 | Heterozygous | c.2442_2445del | p.(Gly817Lyfs*2) | Vervoort et al. 2000 |
| **Unknown status** | | | | | | |
| 7 | F | 38 | Heterozygous | c.2719G>T | p.(Glu907*) | Ayyagari et al. 2002 |
| 8 | F | 30 | Heterozygous | c.2236_2237del | p.(Glu746Argfs*23) | Vervoort et al. 2000 |
| 9 | F | 63 | Heterozygous | c.2236_2237del | p.(Glu746Argfs*23) | Vervoort et al. 2000 |
| 10 | F | 19 | Heterozygous | c.2527del | p.(Glu843Glyfs*246) | Schlottmann et al. 2023 |

**Supplementary Materials and Methods**

***Targeted NGS gene panel***

The panel includes 230 genes of inherited retinal dystrophies (see below). The target regions were comprised of the coding exons and their flanking intronic regions. Capture oligonucleotide probes were designed using the HaloPlex Target Enrichment System (Agilent Technologies Inc., Santa Clara, CA, USA) according to the manufacturer’s protocol. DNA libraries were sequenced on a NovaSeq 6000 sequencer (Illumina Inc., San Diego, CA, USA) (paired-end sequencing 2x150 bases, 96 libraries per lane). Reads were aligned to the Genome Reference Consortium human genome build 19 (GRCh37), DRAGEN (v2023.12.r1) was used to call the single nucleotide variants (SNVs). Variants were annotated the in-house pipeline ANATOLE2.

***CNV detection***

The detection of CNVs was performed by a quantitative analysis of the data obtained from the BAM files. For each patient and for each target (i.e., exon), we calculated the ratio as follows: the depth of the reads for the target divided by the depth of the reads for all targets, and this ratio was divided by the full mean coverage for all control samples analysed on the same NGS run. A ratio value of 1 indicated that the copy number was identical to that of the control samples, and a ratio value of 0.5 revealed only one copy of the allele and a heterozygous deletion. When detected, CNV were confirmed by quantitative PCR (qPCR) using an ABI PRISM 7900 HT instrument (Applied Biosystems) in triplicate for each sample, according to the manufacturer’s protocol.

***List of the 230 IRD genes analyzed by initial short-read sequencing***

***ORF15 Sanger sequencing conditions***

A 2,100 bp region is amplified using the primers below:

E15Flr AGCCAGACAGTTACATGGAAGGTGCAA

E15Rlr TGTCTTTGGCTCCTTAACACAGCTGCATCAG

The sequencing primers are as follows:

ORF15_R4Seq CTCTCCTTCCTCCTTTTCAC

ORF15_R5Seq ACTGGCCATAATCGGGTCACAT

ORF15_R7bSeq CCTTCCTCCTCTTCCCCCTCA

ORF15_R8bSeq TCCTTCCTCCTCTTCCCCCTCCCA

ORF15_R9Seq CCCTGTGTGTTAGTAACTGAC

ORF15_F10Seq GACCTCTGCTCTTTCCCATTTCC

Conditions for primers R4Seq, R5Seq and R9Seq:

96°C 1 min

96°C 20 sec 30 cycles

56°C 10 sec

60°C 4 min

Conditions for primers R7bSeq:

96°C 1 min

96°C 20 sec 30 cycles

58°C 10 sec

63°C 4 min

Conditions for primers R8bSeq and F10Seq:

96°C 1 min

96°C 20 sec 30 cycles

63°C 10 sec

68°C 4 min
